# Multiband linear and circular polarization rotating metasurface based on multiple plasmonic resonances for C, X and K band applications

**DOI:** 10.1038/s41598-020-75081-x

**Published:** 2020-10-22

**Authors:** M. Ismail Khan, Yixiao Chen, Bin Hu, Naeem Ullah, Syed Hashim Raza Bukhari, Shahid Iqbal

**Affiliations:** 1 School of Optics and Photonics, Beijing Institute of Technology, Beijing, 100081 China; 2 Department of Electrical and Computer Engineering, COMSATS University Islamabad, Attock Campus, Islamabad, Pakistan; 3 School of Information Science and Engineering, State Key Laboratory of Millimeter Waves, Southeast University Nanjing, Nanjing, 210096 China

**Keywords:** Engineering, Optics and photonics, Physics

## Abstract

In this work, a multiband polarization converting metasurface is presented which achieves cross-polarization conversion in five frequency bands while linear-to-circular and circular-to-linear polarization transformation in eight frequency bands. The polarization transforming functionality of the structure is spread over an ultra-wide frequency range (5–37 GHz) covering most of X, C, Ku, K and Ka bands. Such an extraordinary ultra-wideband operation originates from multiple plasmonic resonances occurring in the structure based on two coupled rectangular split-ring resonators. Moreover, the polarization transforming capability is stable within the frequency range 5–19 GHz for wide oblique incidence angles, which is up to 60°, both for transverse-electric and transverse-magnetic polarizations. Furthermore, the proposed structure acts as a meta-mirror which preserves handedness of the circular polarization upon reflection. Measurements performed on the fabricated metasurface are found to be consistent with numerical simulation results. The ability to perform three functionalities through a single compact structure with extraordinary wideband, qualifies the proposed design to be a promising candidate for integration with important microwave applications such as satellite, radar, and 5G communication.

## Introduction

Metamaterials offer the freedom to be engineered for desirable electromagnetic (EM) responses and functionalities which were previously considered impossible through conventional materials. A flat metamaterial structure, called metasurface^1^, has succeeded in attracting an overwhelming interest due to its obvious advantages over bulky metamaterials such as simpler structure, easier fabrication, and less cost. The subwavelength unit cells, called meta-atoms, can be designed for enhanced optical activity, even at large wavelengths such as microwaves, to control phase, amplitude, and polarization of the EM waves over much shorter distances when compared to conventional optical materials. Due to a vital role of polarization in many applications such as antennas, satellite communication, radar, contrast imaging, etc., researchers have come up with different metasurface-based designs to manipulate the polarization of the electromagnetic waves.

Literature review shows that the polarization plane can be rotated through the use of anisotropic^2–6^ and chiral metasurfaces, which may be provided with either intrinsic^7–9^ or extrinsic^10,11^ chirality. In this regard, researchers have realized metasurface designs to manipulate the polarization of the impinging wave either in transmission^12–15^ or reflection mode^16–18^. Many cross-polarization conversion metasurfaces, which rotate the plane of polarization by 90°, have been reported^19–28^. In addition to the microwave frequency regime^29–31^, the developments in fabrication technology have pushed the control over polarization to even smaller wavelengths such as terahertz^32–34^, infrared and visible^35–37^ frequencies.

A wideband (2–3.5 GHz) 90°-polarization-rotating metasurface was realized in 2017 for operation in the reflection mode^19^. Then the polarization rotating capability was extended to a wider frequency band (9.1–12.9 GHz) by demonstrating three plasmonic resonances^20^. To further enhance the bandwidth, a double head-arrow structure was used to achieve four plasmonic resonances giving ultra-wideband (6.2–23.4 GHz) polarization rotation^21^. In addition to the 90°-polarization-rotation, i.e. the cross-polarization conversion (CPC), anisotropy of the unit cell can be optimized to achieve linear-to-circular (LTC) polarization conversion and vice versa. In this regard, an LTC converting anisotropic metasurface operating at 2.5 GHz has been reported^38^. The circular polarization conversion functionality was extended to dual bands, 5.50–8.94 GHz and 13.1–15.5 GHz, by using metasurface composed of metallic rectangular loops^39^. Similarly, using a bilayer arrangement of metallic gratings^40^, linear-to-circular conversion has been realized over the frequency range of 11.4–14.3 GHz.

Although, the designs discussed so far can manipulate the polarization of the EM waves, they realized only one type of functionality: either CPC or LTC conversion. A multifunctional metasurface^41^ that can manipulate both linear and circular polarizations over a wide frequency range is highly desirable, as it can reduce the complexity and cost of the whole system. Recently, both linear and circular polarization conversions were realized through a single multifunctional design^42^, however, these functionalities are demonstrated only for normal incidence. To overcome the normal incidence limitation, a structure has been proposed, which can realize both linear and circular polarization conversions in the transmission mode, with large angular stability only for LTC conversion^43^. Besides the polarization conversion, a kind of meta-mirror, by which the handedness of a circularly polarized wave is preserved, has been investigated in several recent research reports^44–46^. The meta-mirror operation has been realized for microwaves in the range of 4.5–6.5 GHz, by using circular split-ring resonators (SRRs)^45^. Similarly, a bilayer meta-mirror has been reported for working in the mid-infrared frequency regime^47^.

In this work, we design and experimentally demonstrate a multifunctional metasurface that can perform three operations: linear-cross polarization conversion, linear-to-circular or circular-to-linear polarization conversion, and meta-mirror operation in an exceptionally ultra-wide frequency range of 5–37 GHz. The designed metasurface is based on coupled rectangular split-ring resonators with different arm widths which resonate at multiple frequencies and hence lead to a multiband operation. Moreover, all the realized three functionalities remain stable for oblique illumination angles up to 60° within the frequency range of 5–19 GHz. Table 1 shows the performance comparison of the proposed design with some already reported works in terms of types of polarization conversion, bandwidth, number of bands, stability against oblique incidence (angular stability), and thickness.

**Table 1:** Performance comparison with other reported works.

References	Type of polarization conversion	Bandwidth (GHz)	No of bands CPC/LTC	Angular stability	Polarization conversion ratio (%)	Thickness (mm)
^17^	CPC	7–19.5	1/0	0°–41.5°	90	3.5
^30^	CPC	8.9–11.1	1/0	0°	99.4	1.27
^49^	CPC and LTC	CPC (6.53–12.07) LTC (13.7–15.6)	1/1	0°	88	0.035
^50^	CPC and LTC	CPC (13.39, 20.29) LTC (14.07–15.71, 17.63–19.55)	2/2	0°	100	1.524
This work	CPC and LTC	CPC (6.3–6.5, 9.1–15.4, 25.3–29, 31–31.8, 35.7–35.9) LTC (6.60–6.67, 8.70–8.95, 15.69–16.25 , 21.35–21.55, 24.15–24.97, 29.52–30.63 , 32.00–32.07, 35.47–35.56)	5/8	0°–60° (5–19 GHz)	100	1.6

## Design and results

**Figure 1:**
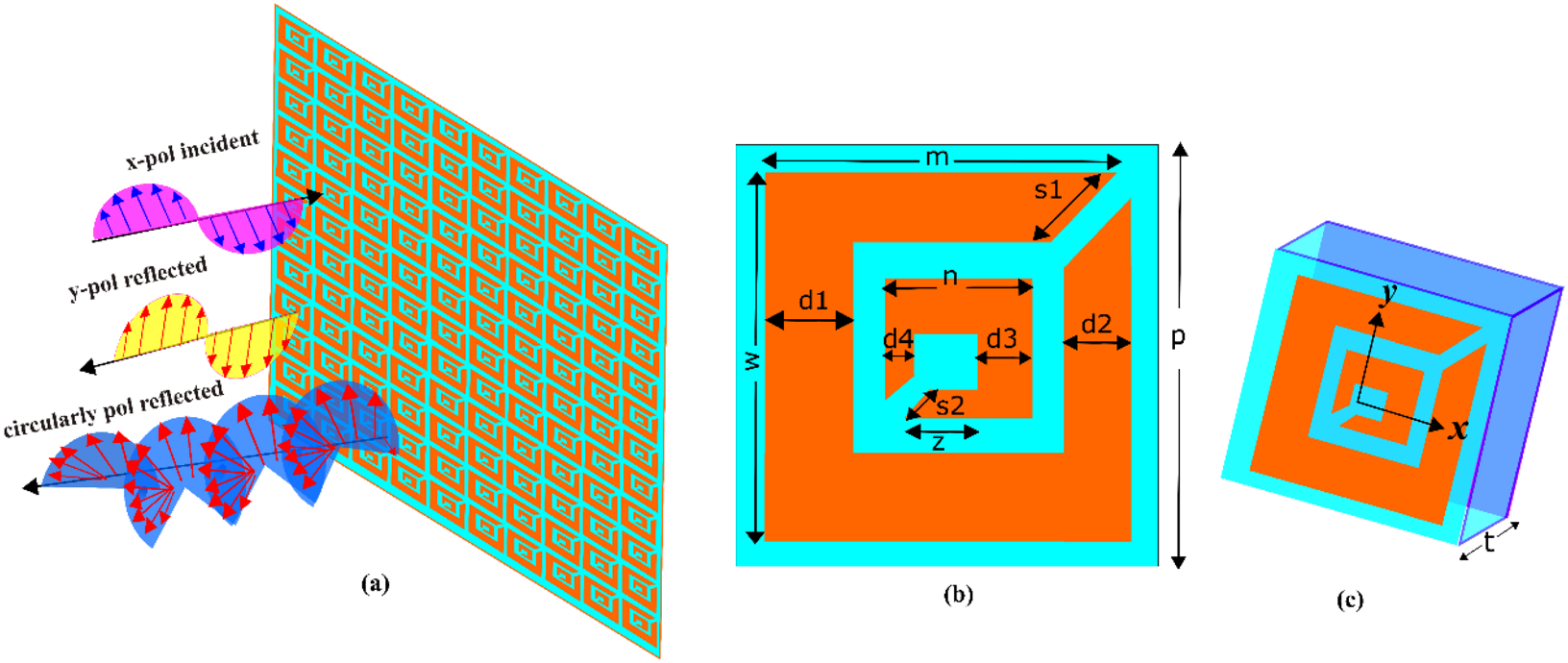
(**a**) Schematic depiction of the proposed multifunctional metasurface. (**b**) Unit cell (**c**) 3D view of the unit cell.

A schematic view of the proposed polarization converting metasurface (PCM) is shown in Fig. 1a. The proposed structure is composed of a dielectric spacer sandwiched by a two-dimensional uniform periodic array of coupled SRRs and a copper reflector. The coupled SRRs functioning as a meta-atom or unit cell, shown in Fig. 1b, comprises of two concentric rings. Each of the rings has a slit positioned at the corner and rotated by 180° with respect to the other. A three-dimensional view of the unit cell is given in Fig. 1c. The physical dimensions of the meta-atom optimized through extensive parametric analysis are: p = 7 mm, w = 6 mm, m = 5.5 mm, n = 2.5 mm, s1 = 1.41 mm, s2 = 0.707 mm, d1 = 1.5 mm, d2 = 1 mm, d3 = 1 mm, d4 = 0.5 mm, z = 1.5 mm, and the thickness of the dielectric spacer is t = 1.6 mm. The material of the SRRs is copper with the conductivity of 5.8 $\times$ 10^7^ S/m, and the dielectric spacer is Rogers with the dielectric constant of 2.33 and the tangent loss of 0.0012.

As the metasurface is backed by a metallic plane, therefore, all the transmission coefficients are zero. Thus, we need to examine only the reflection characteristics of the metasurface. The reflected fields,$\left[ {E_{rx} E_{ry} } \right]^{T}$, are related to the incident fields, $\left[ {E_{ix} E_{iy} } \right]^{T}$, through Jones matrix in Cartesian coordinate system:

1 $$ \left[ {\begin{array}{*{20}c} {E_{rx} } \\ {E_{ry} } \\ \end{array} } \right] = \left[ {\begin{array}{*{20}c} {R_{xx} } & {R_{xy} } \\ {R_{yx} } & {R_{yy} } \\ \end{array} } \right]\left[ {\begin{array}{*{20}c} {E_{ix} } \\ {E_{iy} } \\ \end{array} } \right], $$

where the reflection matrix has complex elements, possessing both magnitude and phase. ${R}_{ij}$ is the reflection coefficient in which the incident linear polarization is denoted by ‘*j*’ while the reflected polarization is denoted by ‘*i*’.

**Figure 2:**
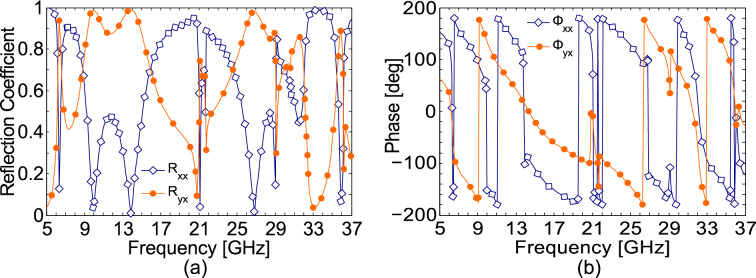
Co- and cross-polarized reflection coefficients for *x*-polarized illumination (**a**) magnitude (**b**) phase.

To study polarization conversion characteristics of the proposed metasurface, we perform numerical simulations using Ansys HFSS. As the metasurface is a periodic structure, therefore, Floquet ports are used where a single unit cell of the structure is placed in the *xy*-plane. EM waves of different polarizations are incident from the top within a frequency range of 5–37 GHz. Figure 2 shows the magnitude and phase results for co- and cross-polarized reflection coefficients when the illuminating wave is *x*-polarized, $E_{i} = \hat{x}E_{o} e^{ikz}$, and normally incident on the metasurface. It is clear from Fig. 2a that the magnitude of the cross-polarized reflection coefficient $\left| {R_{yx} } \right|$ is larger than 0.8 in five frequency bands: 6.3–6.5 GHz, 9.1–15.4 GHz, 25.3–29 GHz, 31–31.8 GHz, and 35.7–35.9 GHz. Strong plasmonic resonances occur at 10 GHz, 14 GHz and 27 GHz where the structure completely rotates the plane of polarization by 90°, i.e., $\left| {R_{yx} } \right| = 1,\left| {R_{xx} } \right| = 0$. Thus, only *y*-polarized wave is reflected for *x*-polarized illumination. As both copper and Rogers can be assumed to be lossless at the microwave frequencies, therefore, by conservation of energy, the reflection coefficients must satisfy $\left| {R_{xx} } \right|^{2} + \left| {R_{yx} } \right|^{2} = 1.$ As expected, the magnitude of the co-polarized reflection co-efficient can be found to be very small in the aforementioned five working bands and it approaches to zero at resonance frequencies. In addition to the amplitude response, the anisotropic nature of the structure causes different phase delays for the reflected co- and cross-polarized fields. This can be seen from Fig. 2b that the phases of the co- and cross reflection coefficients, $\Phi_{xx}$ and $\Phi_{yx}$ are different in most of the frequency bands. Although, we have presented simulation results only for the *x*-polarized waves, the response for the *y*-polarized illumination can be found from reciprocity. Since, the metasurface is lossless and does not possess any non-reciprocal element in the structure, time reversal symmetry is maintained, which ensures that response of the structure for *y*-polarized illumination is the same as for *x*-polarization. Therefore, co- and cross-polarized reflection coefficients for *y*-polarized impinging wave are similar to those of *x*-polarization, *R*_*xx*_ = *R*_*yy*_ and *R*_*yx*_ = *R*_*xy*._ This can be also understood from the observation that the unit cell structure is the same along both *x*- and *y*-axis, thus it gives similar electromagnetic response to both *x*- and *y*-polarized illuminations.

The cross-polarization conversion functionality of an electromagnetic structure is better captured by defining the polarization conversion ratio (PCR) which is the ratio of the power reflected in the cross-polarized component to the total reflected power, given mathematically by:

2 $$ PCR = \frac{{\left| {R_{yx} } \right|^{2} }}{{\left| {R_{yx} } \right|^{2} + \left| {R_{xx} } \right|^{2} }}. $$

**Figure 3:**
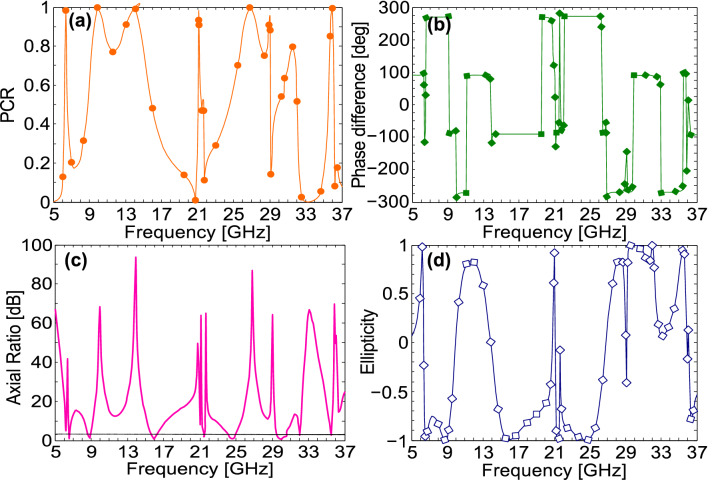
(**a**) Polarization conversion ratio (**b**) Phase difference between co- and cross-polarized fields (**c**) Axial ratio (**d**) Ellipticity.

It is obvious from the PCR results presented in Fig. 3a that within the CPC bands of 6.3–6.5 GHz, 9.1–15.4 GHz, 25.3–29 GHz, 31–31.8 GHz, and 35.7–35.9 GHz, PCR is larger than 75% and approaches 100% at the resonance frequencies.

In order to study linear-to-circular polarization conversion functionality of the metasurface, it is important to recall that ideally, for circular polarization, the amplitudes of the two mutually orthogonal fields must be the same, while their phase difference should be odd multiple of 90°, i.e. $\Delta \Phi \, = n\pi /2$, where *n* is an odd integer. The phase difference between co- and cross-polarized reflection coefficients is given in Fig. 3b. The effect of both amplitudes and phase difference of the reflected fields is taken into consideration by the axial ratio (AR) which is given by:

3 $$ AR = \frac{1}{2}\left\{ {\frac{{\left| {R_{xx} } \right|^{2} + \left| {R_{yx} } \right|^{2} + \left( {\left| {R_{xx} } \right|^{4} + \left| {R_{yx} } \right|^{4} + 2\left| {R_{xx} } \right|^{2} \left| {R_{yx} } \right|^{2} \cos \left( {2\Delta \varphi } \right)} \right)^{\frac{1}{2}} }}{{\left| {R_{xx} } \right|^{2} + \left| {R_{yx} } \right|^{2} - \left( {\left| {R_{xx} } \right|^{4} + \left| {R_{yx} } \right|^{4} + 2\left| {R_{xx} } \right|^{2} \left| {R_{yx} } \right|^{2} \cos \left( {2\Delta \varphi } \right)} \right)^{\frac{1}{2}} }}} \right\}^{\frac{1}{2}} . $$

where we have used the fact that, the amplitude of the incident field ($$E_{iox} )$$ is normalized, $$E_{iox} = 1$$, therefore, $$R_{xx} = E_{oxr}$$ and $$R_{yx} = E_{oyr}$$ where $$E_{oxr}$$ and $$E_{oyr}$$ are the amplitudes of the *x* and *y* components of the reflected electric field. The axial ratio represents circular polarization if it is less than or equal to 3 dB, i.e. *AR* ≤ 3 dB^38^. The axial ratio obtained from Eq. (3) is presented in Fig. 3c. It is clear from Fig. 3c that the criterion for circular polarization (*AR* ≤ 3 dB) is satisfied within eight frequency bands: 6.60–6.67 GHz, 8.70–8.95 GHz, 15.69–16.25 GHz, 21.35–21.55 GHz, 24.15–24.97 GHz, 29.52–30.63 GHz, 32.00–32.07 GHz, and 35.47–35.56 GHz. Thus, the proposed design exhibits linear-to-circular polarization conversion within the eight frequency bands. To examine handedness of the reflected circular polarization, we make use of Stokes’ parameters^48^ which are defined as:

4 $$ \begin{gathered} S_{0} = \left| {R_{xx} } \right|^{2} + \left| {R_{yx} } \right|^{2} \hfill \\ S_{1} = \left| {R_{xx} } \right|^{2} - \left| {R_{yx} } \right|^{2} \hfill \\ S_{2} = 2\left| {R_{xx} } \right|\left| {R_{yx} } \right|\cos \Delta \varphi \hfill \\ S_{3} = 2\left| {R_{xx} } \right|\left| {R_{yx} } \right|\sin \Delta \varphi . \hfill \\ \end{gathered} $$

Normalized ellipticity is defined as $e={S}_{3}/{S}_{0}$. It can be easily deduced from the Stokes’ parameters that the normalized ellipticity is + 1 for right-handed circularly polarization (RHCP) and − 1 for left-handed circular polarization (LHCP). The results presented in Fig. 3d show that the ellipticity is 1 and hence the reflected EM wave is RHCP in three frequency bands: 29.52–30.63 GHz, 32.00–32.07 GHz, and 35.47–35.56 GHz. On the other hand, an *x*-polarized linear wave is reflected as LHCP, in five frequency bands: 6.60–6.67 GHz, 8.70–8.95 GHz, 15.69–16.25 GHz, 21.35–21.55 GHz and 24.15–24.97 GHz.

It is also important to investigate response of the metasurface under circularly polarized illumination. As we have already obtained the reflection magnitude and phase response of the metasurface in rectangular basis, therefore, we can easily transform these responses into circular basis using:

5 $$ {\mathbf{R}}_{{{\mathbf{CP}}}} = \left( {\begin{array}{*{20}c} {R_{rr} } & {R_{rl} } \\ {R_{lr} } & {R_{ll} } \\ \end{array} } \right) = \frac{1}{2}\left( {\begin{array}{*{20}c} {R_{xx} - R_{yy} - i\left( {R_{xy} + R_{yx} } \right)} & {R_{xx} + R_{yy} + i\left( {R_{xy} - R_{yx} } \right)} \\ {R_{xx} + R_{yy} - i\left( {R_{xy} - R_{yx} } \right)} & {R_{xx} - R_{yy} + i\left( {R_{xy} + R_{yx} } \right)} \\ \end{array} } \right), $$

where the subscripts ‘*r*’ and ‘*l*’ represent RHCP and LHCP, respectively. ${R}_{rl}$ shows reflection coefficient when the incident wave is LHCP and the reflected wave is RHCP. A normal metallic reflector or mirror reflects RHCP incident wave as LHCP wave and vice versa. Interestingly, it can be deduced from Eq. (5) and the results shown in Fig. 2a,b respectively, that the proposed design is able to reflect an RHCP wave as RHCP with ${R}_{rr}\approx 1$, and LHCP as LHCP with ${R}_{ll}\approx 1$ in five CPC frequency bands. This polarization maintaining capability is captured by defining polarization maintaining ratio (PMR) for circular polarization as:

6 $$ PMR = \frac{{\left| {R_{rr} } \right|^{2} }}{{\left| {R_{lr} } \right|^{2} + \left| {R_{rr} } \right|^{2} }}. $$

It is easy to show that the PMR for circular polarization becomes equal to PCR in five CPC frequency bands as $\left| {R_{rr} } \right| = \left| {R_{ll} } \right| = \left| {R_{yx} } \right| = \left| {R_{xy} } \right|$ while $\left| {R_{lr} } \right| = \left| {R_{rl} } \right| = \left| {R_{xx} } \right| = \left| {R_{yy} } \right| \approx 0$ . Thus, the proposed structure functions as a meta-mirror, which unlike conventional mirrors, preserves handedness of the circular polarization upon reflection in frequency bands: 6.3–6.5 GHz, 9.1–15.4 GHz, 25.3–29 GHz, 31–31.8 GHz, and 35.7–35.9 GHz.

It is also important to note from Eq. (6) that in the eight circular polarization conversion bands the designed PCM converts a circularly polarized wave into linearly polarized wave, as $\left| {R_{rr} } \right| = \left| {R_{ll} } \right| = 0$, while $\left| {R_{lr} } \right| = \left| {R_{rl} } \right| = 1$. This can be also deduced from the reciprocity of the metasurface due to which time reversal symmetry is not broken. Since the proposed metasurface performs both LTC and CP-to-LP conversions, therefore, it also functions as a quarter-wave plate^46^ (QWP) operating in the reflection mode.

**Figure 4:**
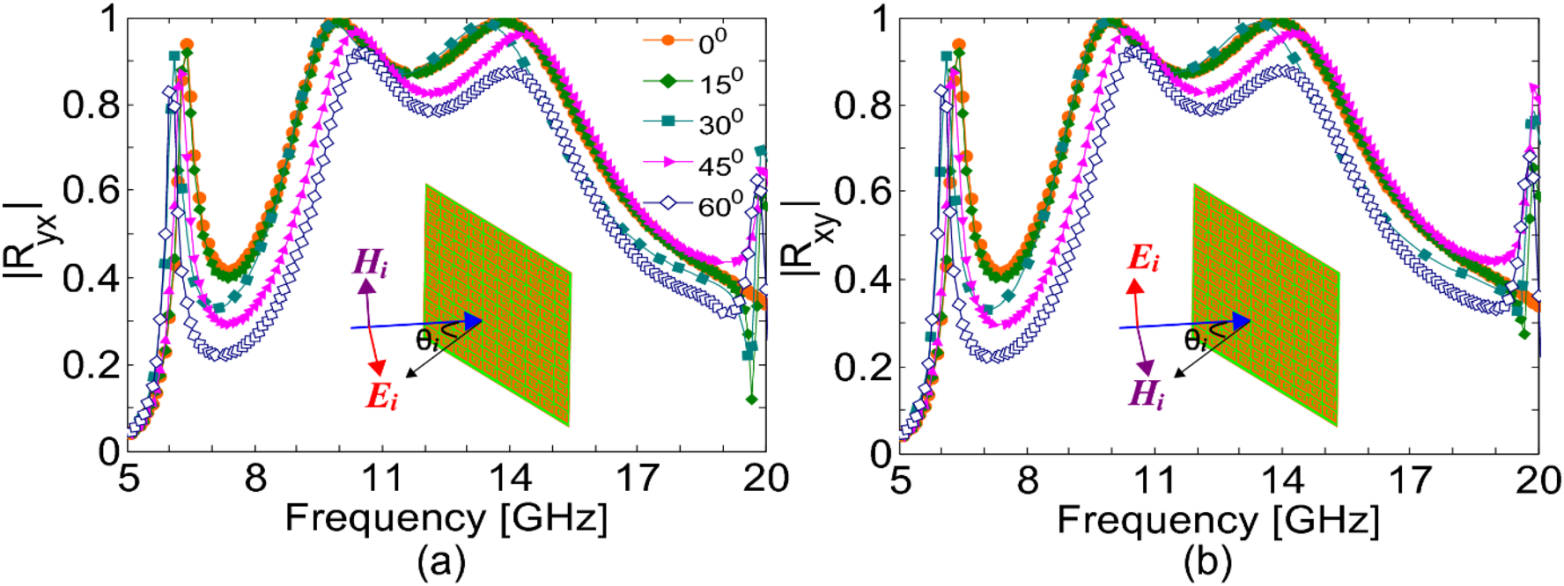
Magnitude of the cross-polarized reflection coefficient under oblique incidence for (**a**) TM-polarization (**b**) TE-polarization.

In most of the practical scenarios, especially in the microwave frequency regime, the impinging EM waves may have arbitrary incidence angles. Therefore, it is of practical interest for the metasurface to respond robustly to the incidence angle. It is important to note that one of the necessary conditions for the angular stability against oblique incidence is the relatively small size of the unit cell compared to the operating wavelength^16^. As the operating bands of our proposed structure are scattered in a wide frequency range 5–37 GHz, therefore, it is not possible to demonstrate angular stability for higher frequencies where the size of the unit cell becomes comparable to the wavelength such as p = 0.87λ at 37 GHz. Therefore, we numerically investigate the oblique incidence response of the metasurface for both transverse electric (TE) polarization (the electric field of the incident wave is in the *yz*-plane) and transverse magnetic (TM) polarization (the electric field of the incident wave is in the *xz*-plane), in the frequency range of 5–20 GHz. It can be seen from Fig. 4 that, for both TM and TE polarizations, the magnitude of the cross-polarized reflection coefficient remains stable against the oblique incidence angle up to 60° in the frequency range of 5–19 GHz. It can be also noted from Fig. 4 that the angular stability decreases at higher frequencies and it is significantly reduced at 20 GHz where the size of the unit cell is 0.46λ. (The angular stability results for the entire frequency sweep, 5–37 GHz, are given in the Supplementary Information).

## Theoretical analysis

The process of polarization conversion through a metasurface can be considered as a linear time-invariant system where the input vector (incident fields) is transformed into some output vector (reflected fields). Equation (1) relates the input and output fields through reflection coefficient matrix, which represents the modulation of the metasurface at a particular frequency. To find eigen-polarization (eigenvector) and eigenvalues at CPC frequencies where *|R*_*yx*_*|* =*|R*_*xy*_*| ≈* 1 and *|R*_*xx*_*|* =*|R*_*yy*_*| ≈* 0, we solve the following matrix equation:

7 $$ RY - mY = 0, $$

where ***Y*** is the eigen-polarization vector, *m* is the eigenvalue, and ***R ***is the reflection coefficient matrix which at CPC frequencies is:

8 $$ {\mathbf{R}} = \left( {\begin{array}{*{20}c} 0 & 1 \\ 1 & 0 \\ \end{array} } \right). $$

**Figure 5:**
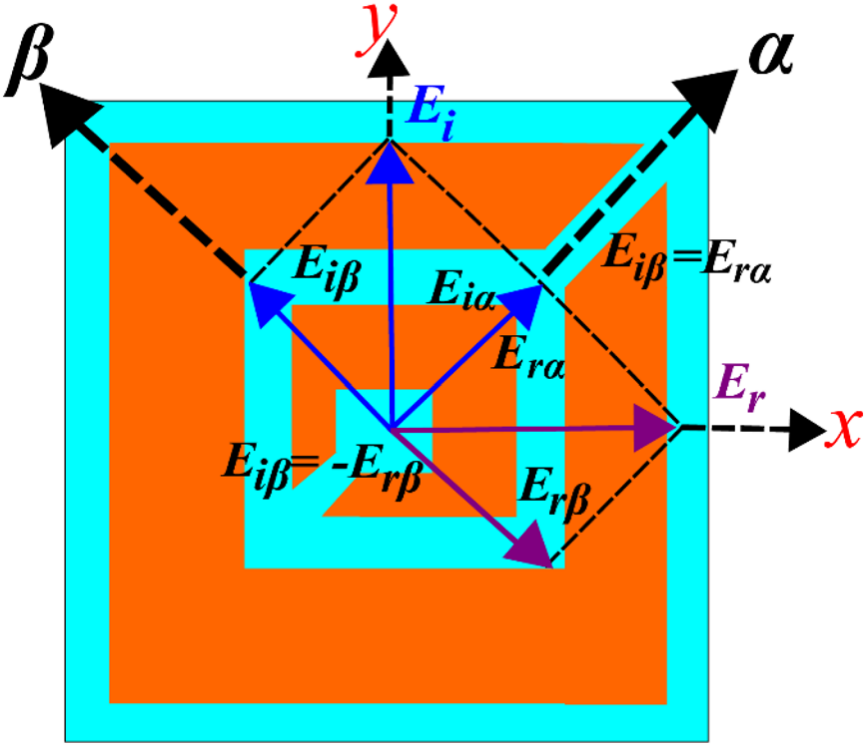
*y*-polarized incident electric field is decomposed into orthogonal components along *α*- and *β*-axis, *α*-component is reflected in phase while *β*-component is reflected out of phase which orients the total reflected field along the *x*-axis.

Solving Eq. (7) gives linearly independent eigenvectors for the polarization rotating metasurface which are ***y***_1_ = ***α*** = (1 1)^*T*^ and **y**_2_ = ***β*** = (− 1 1)^*T*^, with eigenvalues *e*^*i*0^ = 1 and, *e*^*iπ*^ = − 1, respectively. Physically this implies that α- and *β*-polarized incident waves (*α*- and *β*-axis are tilted at ± 45° to the *y*-axis, as shown in Fig. 5) are reflected by the metasurface with magnitude of 1 and phase of 0° and 180°, respectively, without any polarization rotation. Since no polarization plane rotation takes place when the impinging wave is *α* or *β*-polarized, therefore, *|R*_*αα*_* |* = *|R*_*ββ*_*| ≈* 1 and *|R*_*βα*_| = *|R*_*αβ*_*| ≈* 0. Keeping this in mind, consider a normally incident *y*-polarized plane EM wave $E_{i} = \hat{y}E_{i} e^{ikz}$ , with wave number *k* as shown in Fig. 5, it can be written as the sum of two orthogonal ***α*** and ***β*** components, $E_{i} = \hat{y}E_{i} = \hat{\alpha }E_{i\alpha } + \hat{\beta }E_{i\beta }$ at *z* = *0*, where $E_{i\alpha } = E_{i\beta } = 0.707E_{i}$.

As the reflected *α* and *β*-polarized components have the same magnitude and phase of 0° and 180°, respectively, the reflected electric field becomes:

9 $$ {\varvec{E}}_{{\varvec{r}}} = \hat{\alpha }E_{r} - \hat{\beta }E_{r} = \hat{x}E_{r} . $$

It can be noted from Eq. (9) that the incident field is reflected along the *x*-axis and hence rotated by 90°. This can be also understood pictorially from Fig. 5 where ***E***_*r*_, obtained from the vector sum of *E*_*rα*_ and *E*_*rβ*_, is along the *x*-axis.

In principle, to achieve CPC, the phase difference between two eigen-polarizations should be odd multiple of 180°, i.e. $\Delta \phi = \phi_{\alpha \alpha } - \phi_{\beta \beta } = n\pi$, irrespective of the actual values of $\phi_{\alpha \alpha }$ and $\phi_{\beta \beta }$. To show this mathematically, a *y*-polarized incident wave is considered, i.e. $E_{i} = \hat{y}E_{i} = \hat{\alpha }E_{i\alpha } + \hat{\beta }E_{i\beta }$ at *z* = 0, where $E_{i\alpha } = E_{i\beta } = 0.707E_{i}$. The reflected electric field is:

10 $$ {\varvec{E}}_{{\varvec{r}}} = \hat{\alpha }R_{\alpha \alpha } E_{i\alpha } e^{{i\varphi_{\alpha \alpha } }} + \hat{\beta }R_{\beta \beta } E_{i\beta } e^{{i\varphi_{\beta \beta } }} , $$

where *|R*_*αα*_* |* =*|R*_*ββ*_*| ≈* 1 and *|R*_*βα*_*|* = *|R*_*αβ*_*| ≈* 0. Therefore, Eq. (10) gives:

$$ {\varvec{E}}_{{\varvec{r}}} = \hat{\alpha }E_{i\alpha } e^{{i\varphi_{\alpha \alpha } }} + \hat{\beta }E_{i\beta } e^{{i\varphi_{\beta \beta } }} . $$

Now, the reflected field is perpendicular to the incident wave if $E_{i} .E_{r} = 0$, which gives:

11 $$ E_{i\alpha } e^{{i\varphi_{\alpha \alpha } }} \left( {1 + e^{i\Delta \varphi } } \right) = 0. $$

Equation (11) has nontrivial solution if and only if $\Delta \varphi = n\pi$, for $n = \pm 1, \pm 3, \pm 5 \ldots$

**Figure 6:**
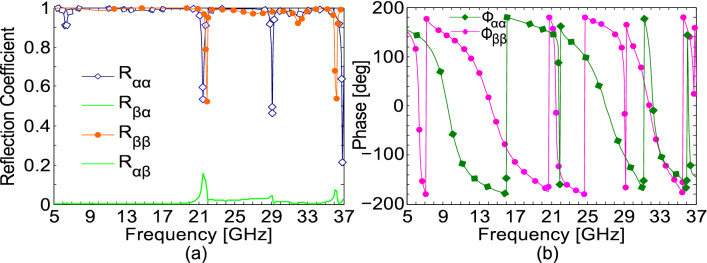
(**a**) Magnitude and (**b**) phase of the reflection coefficients for *α-* and *β*-polarization.

To verify the above theoretical analysis, numerical simulations were conducted for the proposed design under α- and β-polarized illumination. As expected, Fig. 6 shows that the magnitude of the cross-polarized reflection coefficients, *|R*_*βα*_* |* and *|R*_*αβ*_*|*, are negligible while the co-polarized reflection coefficients, *|R*_*αα*_* |* and *|R*_*ββ*_*|*, have magnitudes approaching 1 for most of the operating frequencies. The phase values for the eigen polarizations, $\phi_{\alpha \alpha }$ and $\phi_{\beta \beta }$, plotted in Fig. 6b, show that the phase difference at CPC frequencies (10 GHz, 14 GHz and 27 GHz) reaches 180°. It is also important to note that the absolute phase is 0° at 10 GHz and 27 GHz for α-polarization, while it is 0° at 14 GHz for β-polarization.

To better understand the process of polarization rotation, we need to elaborate on the physical mechanism working behind the conversion. The electric and magnetic fields of the illuminating wave interact with the artificial meta-atoms of the metasurface and thus polarize them electrically and magnetically. This effectively results in electric and magnetic dipole moments in the meta-atoms where each of the two is coupled to both electric and magnetic fields due to the bi-anisotropy of the SRR. The spatially averaged effective dipole moments are related to the illuminating fields as:

12 $$ \left[ {\begin{array}{*{20}c} p \\ m \\ \end{array} } \right] = \left[ {\begin{array}{*{20}c} {\wp_{ee} } & {\wp_{em} } \\ {\wp_{me} } & {\wp_{mm} } \\ \end{array} } \right]\left[ {\begin{array}{*{20}c} E \\ H \\ \end{array} } \right], $$

where $p = \left[ {p_{{{\mathbf{\mathcal{X} }}}} ,p_{{\mathbf{\mathcal{Y}}}} } \right]^{{\rm T}}$ and $m = \left[ {m_{x} ,m_{y} } \right]^{{\mathbf{\rm T}}}$ show electric and magnetic dipole moments and $E = [E_{x} ,E_{Y} ]^{T}$, $H = [H_{x} ,H_{Y} ]^{T}$ represent electric and magnetic fields while $\wp_{em}$ stands for electric–magnetic polarizability. The electric and magnetic dipole moments of the meta-atoms determine the effective surface impedance, given by $Z_{s} \left( \omega \right) = \sqrt {{{\mu_{s} \left( \omega \right)} \mathord{\left/ {\vphantom {{\mu_{s} \left( \omega \right)} {\varepsilon_{s} \left( \omega \right)}}} \right. \kern-\nulldelimiterspace} {\varepsilon_{s} \left( \omega \right)}}}$, where $\mu_{s} \left( \omega \right)$ and $\varepsilon_{s} \left( \omega \right)$ are frequency-dependent magnetic permeability and electric permittivity respectively. This surface impedance is used to find the frequency dependent reflection coefficient $R\left( \omega \right)$ for normal incidence, which is calculated by:

13 $$ R\left( \omega \right) = \frac{{Z_{s} \left( \omega \right) - Z_{o} }}{{Z_{s} \left( \omega \right) + Z_{o} }} $$

in which ${Z}_{o}$=377Ω is the impedance of the free space. It is obvious from Eq. (13) that, $R=1$ when the surface impedance of the metasurface is much higher than the impedance of the free space, ${Z}_{s}({\omega }_{r})\gg {Z}_{o}$, where ${\omega }_{r}$ is the resonance frequency. At the frequencies where such condition is satisfied, the metasurface is called high impedance surface (HIS) which unlike a conventional reflector, reflects the incident EM waves with unity magnitude and 0° phase reversal. As discussed previously, when one component of the incident field is reflected with phase of 0° while the other orthogonal component is reflected with phase of 180°, the polarization plane of the incident wave will be rotated by 90°. This means that the metasurface should behave as HIS for one component while as ordinary reflector for the other component. To see whether HIS condition is achieved by the proposed design, we investigate surface currents on the metasurface which are generated by the time changing electric and magnetic dipole moments induced by the time harmonic incident fields:

14 $$ \left[ {\begin{array}{*{20}c} {J_{s} } \\ {M_{s} } \\ \end{array} } \right] = i\omega \left[ {\begin{array}{*{20}c} {\wp_{ee} } & {\wp_{em} } \\ {\wp_{me} } & {\wp_{mm} } \\ \end{array} } \right]\left[ {\begin{array}{*{20}c} E \\ H \\ \end{array} } \right]. $$

**Figure 7:**
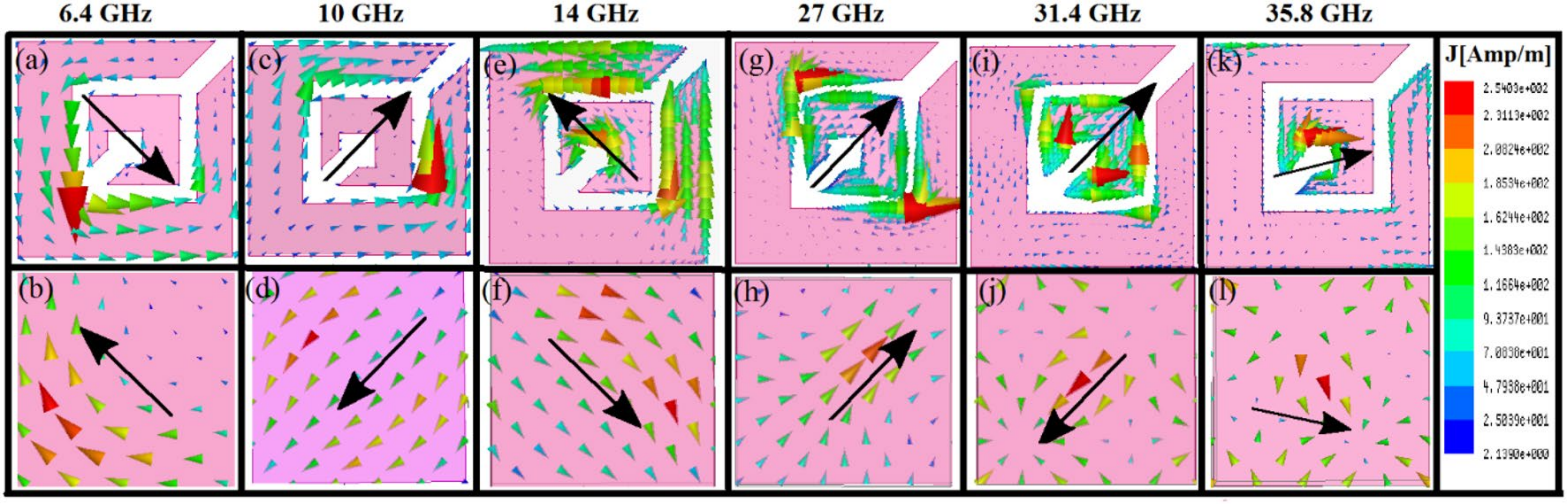
Surface currents at top (**a**, **c**, **e**, **g**, **i**, **k**) and bottom (**b**, **d**, **f**, **h**, **j**, **l**) layer at 6.4 GHz (**a**, **b**), 10 GHz (**c**, **d**), 14 GHz (**e**, **f**), 27 GHz (**g**, **h**), 31.4 GHz (**i**, **j**) and 35.8 GHz (**k**, **l**). The large black arrow represents the vector sum of the surface currents.

Surface current distributions obtained from numerical simulations are shown in Fig. 7, which are at CPC frequencies of 6.4 GHz, 10 GHz, 14 GHz, 27 GHz, 31.4 GHz, and 35.8 GHz respectively. It can be seen from Fig. 7 that resonances occurring at 6.4 GHz, 10 GHz, 14 GHz, and 31.4 GHz are magnetic in nature where the vector sum of currents, represented by the black arrow, at the top and bottom layers are anti-parallel which significantly enhances the magnetic field in the dielectric spacer region. The strong magnetic flux increases the effective magnetic permeability which makes surface impedance much larger than free space impedance thus achieving HIS condition, $\mathrm{Z}({\omega }_{r})\gg {Z}_{o}$, which leads to reflection coefficient with unity magnitude and 0^º^ phase. The resonances occurring at 27 GHz and 35.8 GHz are electric in nature where the top and bottom layer currents are parallel, though, they are not completely parallel in the case of 35.8 GHz giving weak CPC which can be seen from the small value of *R*_*yx*_ at 35.8 GHz in Fig. 2a.

## Experimental verification

**Figure 8:**
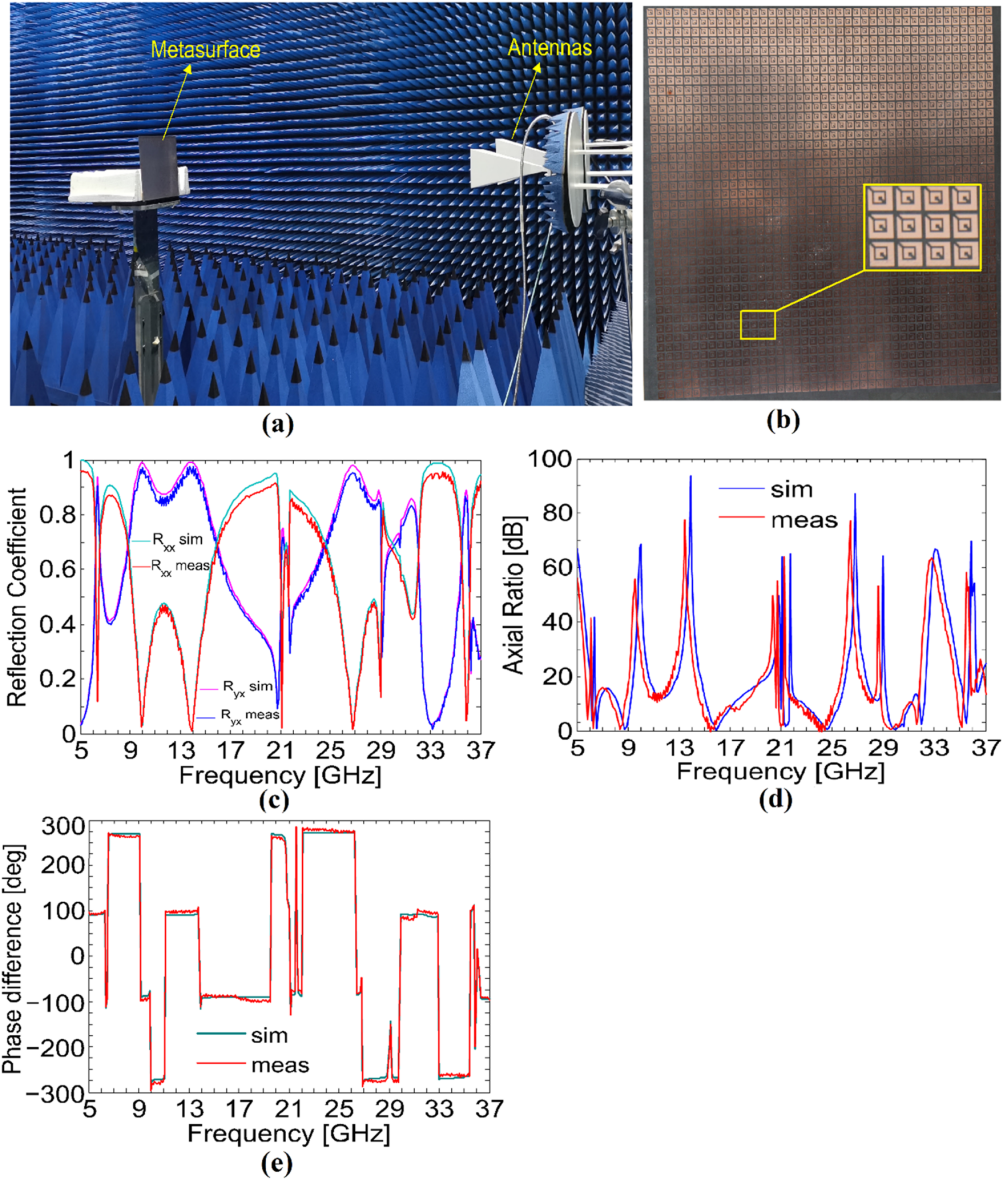
(**a**) Measurement setup (**b**) Fabricated sample (**c**) Magnitude of the simulated and measured co- and cross-polarized reflection coefficients. (**d**) Axial ratio (**e**) Phase difference.

To verify the numerical simulation results through experimental measurements, we fabricated a prototype of the proposed polarization rotator on 270 × 270 × 1.6 mm^3^ Rogers sheet. The prototype, shown in Fig. 8b, is prepared through well known PCB techniques, where 38 × 38 unit cells were etched out on one side of the sheet while the metallic cladding on the other side is left unchanged. To measure co- and cross-polarized reflection coefficients, the fabricated sample is placed in front of two horn antennas, where one antenna transmits while the other receives the EM waves.

The vector network analyzer is connected to antennas to get the amplitude and phase of the signal reflected from the metasurface. To avoid unwanted reflections, the whole measurement setup, shown in Fig. 8a is established inside a fully anechoic chamber. Due to exceptionally ultra-wide frequency range, several antennas were used to get measurements for the entire operating frequency band. In order to measure co-polarized reflection coefficients, the two antennas are placed along the same orientation, either horizontal or vertical, while they are oriented perpendicular to each other for measuring cross-polarized reflection coefficient. Before doing measurements for the actual metasurface, we measured reflection coefficients for a simple metallic plane with the same dimensions as those of the fabricated sample for comparison purpose. The measured and simulated results obtained for the *x*-polarized incident wave are presented in Fig. 8c–e for the magnitude of co- and cross-polarized reflection coefficient (Fig. 8c), axial ratio (Fig. 8d), and phase difference (Fig. 8e). It can be seen from Fig. 8c–e that the measured and the simulated results are in good agreement. The small differences between the measured and simulated results are caused by fabrication imperfections and the finite size of the fabricated sample which causes diffraction effects.

## Conclusion

In summary, we have realized an ultrathin multiband polarization converting metasurface which achieves cross-polarization conversion in five frequency bands: 6.3–6.5 GHz, 9.1–15.4 GHz, 25.3–29 GHz, 31–31.8 GHz, and 35.7–35.9 GHz while linear-to-circular and circular-to-linear polarization transformation in eight frequency bands: 6.60–6.67 GHz, 8.70–8.95 GHz, 15.69–16.25 GHz, 21.35–21.55 GHz, 24.15–24.97 GHz, 29.52–30.63 GHz, 32.00–32.07 GHz, and 35.47–35.56 GHz. The proposed design is based on two coupled rectangular split-ring resonators which gets multiple plasmonics resonances enabling the metasurface to achieve polarization transformation over an ultra-wide frequency range (5–37 GHz) covering most of X, C, Ku, K and Ka bands. Moreover, it is shown that the proposed design preserves handedness of the circular polarization in the frequency regimes where cross-polarization conversion occurs. Furthermore, all the three functionalities of the metasurface are robust to variations in the incidence angle for frequency range 5–19 GHz. The physical mechanism behind the polarization transformation is elucidated through surface current distribution. All the numerical simulation results are verified through experimental measurements.

## Methods

Numerical simulations of the design are conducted using Ansys HFSS. The periodicity of the structure in the *xy*-plane is ensured by applying Floquet ports. The designed structure is placed in the *xy*-plane and EM waves of different polarizations are incident from the top port from which scattering parameters (S-parameters) are obtained. For experimental characterization, the metasurface is fabricated by printed-circuit-board (PCB) techniques using 270 × 270 × 1.6 mm^3^ Rogers sheet. The fabricated metasurface is placed in front of two horn antennas at a distance far enough so that near field effects are avoided. The antennas are connected with Agilent vector network analyzer N5232A through co-axial cable. Before doing measurements for actual metasurface, S-parameters for a simple copper reflector with the same dimensions as metasurface are obtained for calibration purpose. The receiving antenna is kept aligned with transmitting antenna for co-polarized reflections while it is rotated relatively by 90° for cross-polarized coefficients. All the measurements are conducted inside anechoic chamber so that unwanted reflections are avoided.

## Supplementary information

Supplementary Information.

## Data Availability

The datasets generated during and/or analyzed during the current study are available from the corresponding author on reasonable request.
