# Supplementary material for: Multiband linear and circular polarization rotating metasurface based on multiple plasmonic resonances for C, X and K band applications
Source: Sci Rep. 2020 Oct 22;10:17981. doi: 10.1038/s41598-020-75081-x (PMC7582135; doi:10.1038/s41598-020-75081-x)
Supplement: Supplementary file 1 — Supplementary Information. [file 41598_2020_75081_MOESM1_ESM.docx]

| **Multiband linear and circular polarization rotating metasurface based on multiple plasmonic resonances for C, X and K band applications** |  |
| --- | --- |

**M. Ismail Khan^1,2^, Yixiao Chen^1^, Bin Hu^1*^, Naeem Ullah^1^, Syed Hashim Raza Bukhari^2^, Shahid Iqbal^3^**

**SUPPLEMENTARY MATERIAL**

The cross-polarized reflection coefficients when the incident wave is obliquely incident on the metasurface are presented in Fig. S1(a) and Fig. S1(b) for |*R_yx_*| and |*R_xy_*| respectively in the frequency range 5-37 GHz. It can be noted from Fig. S1 that the angular stability decreases at higher frequencies and it is significantly reduced at 20 GHz where the size of the unit cell is 0.46λ. The angular stability is further deteriorated with increasing frequencies beyond 20 GHz. This deterioration in the response of the metasurface at oblique incidence for higher frequencies is caused by the increase in the relative size of the unit cell compared to the wavelength which is 0.87λ at 37 GHz.

*
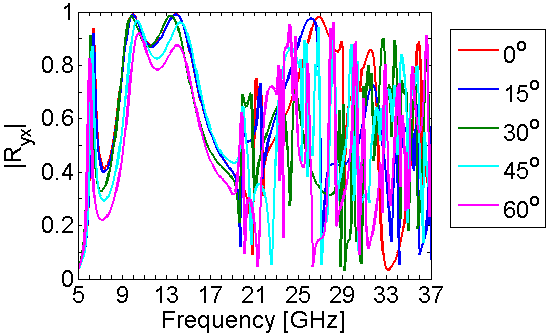
*

Fig. S1(a)

*
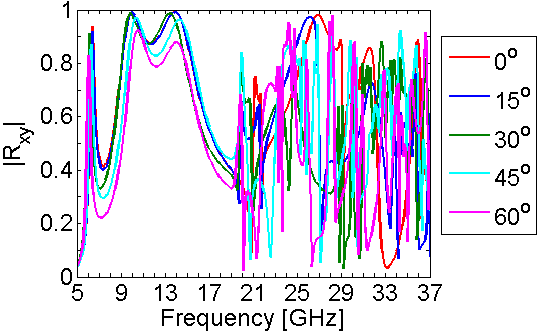
*

Fig. S1(b)
